# Supplementary material for: Gestational Age-Dependent Regulation of Transthyretin in Mice during Pregnancy
Source: Biology (Basel). 2023 Jul 26;12(8):1048. doi: 10.3390/biology12081048 (PMC10451295; doi:10.3390/biology12081048)
Supplement: Supplementary file 1 [file biology-12-01048-s001.zip › biology-2499049-supplementary.pdf]

RT-PCR raw data

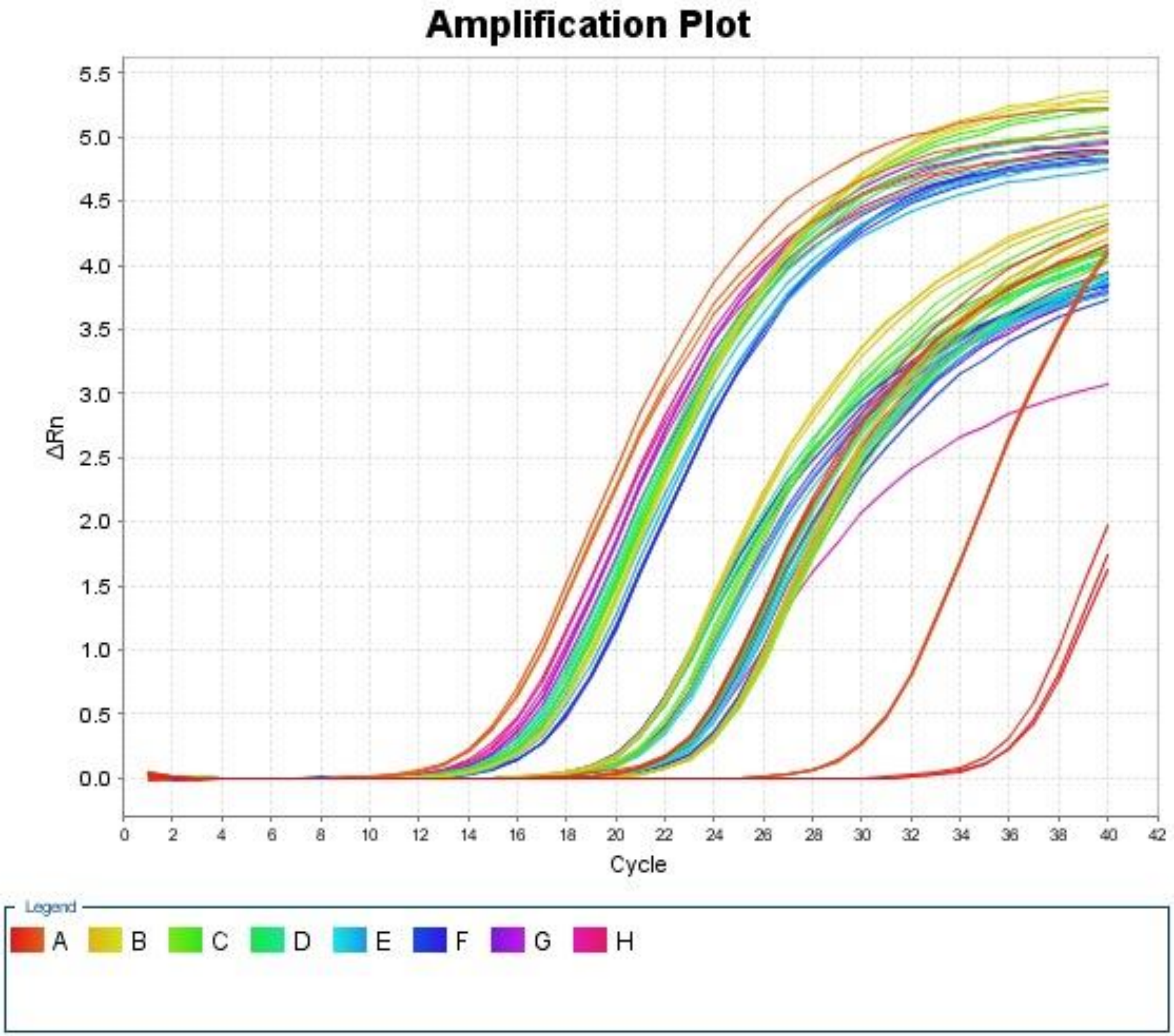

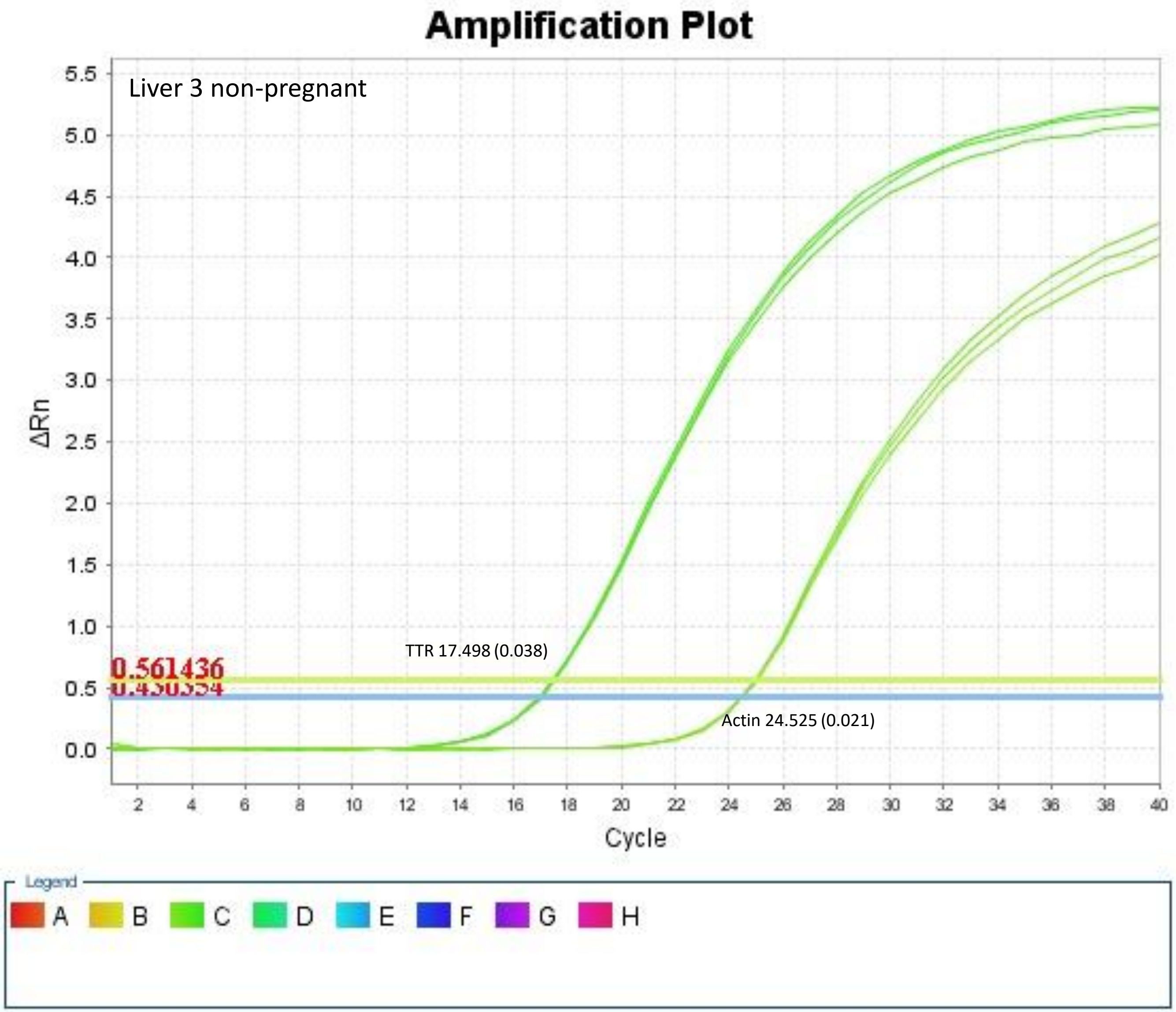

Amplification Plot
Liver 3 non-pregnant
ΔRn
5.5
5.0
4.5
4.0
3.5
3.0
2.5
2.0
1.5
1.0
0.5
0.0
0.561436
TTR 17.498 (0.038)
Actin 24.525 (0.021)
2 4 6 8 10 12 14 16 18 20 22 24 26 28 30 32 34 36 38 40
Cycle
Legend
A B C D E F G H

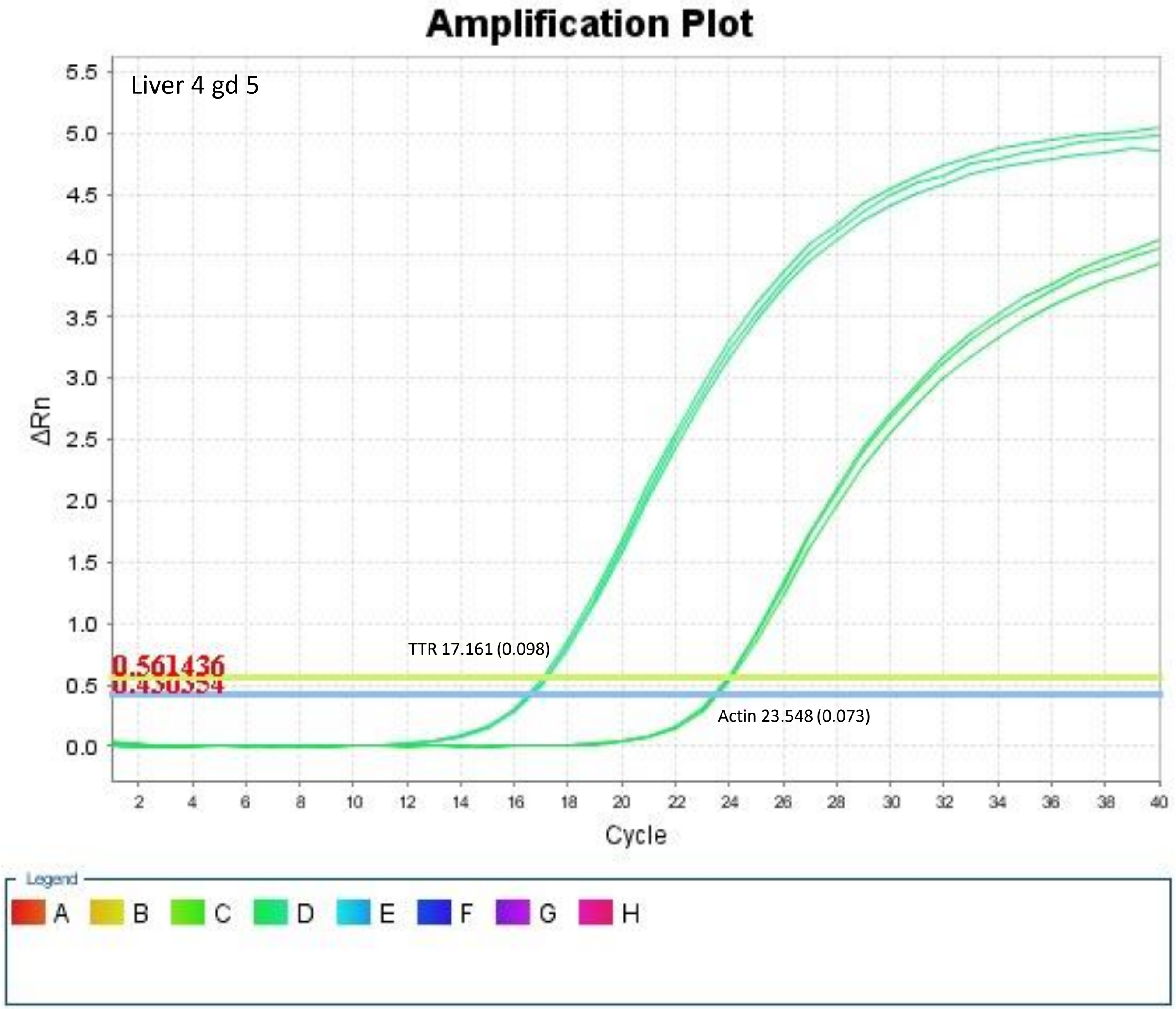
Amplification Plot
Liver 4 gd 5
0.561436
TTR 17.161 (0.098)
Actin 23.548 (0.073)
ΔRn
Cycle
5.5
5.0
4.5
4.0
3.5
3.0
2.5
2.0
1.5
1.0
0.5
0.0
2
4
6
8
10
12
14
16
18
20
22
24
26
28
30
32
34
36
38
40
Legend
A
B
C
D
E
F
G
H

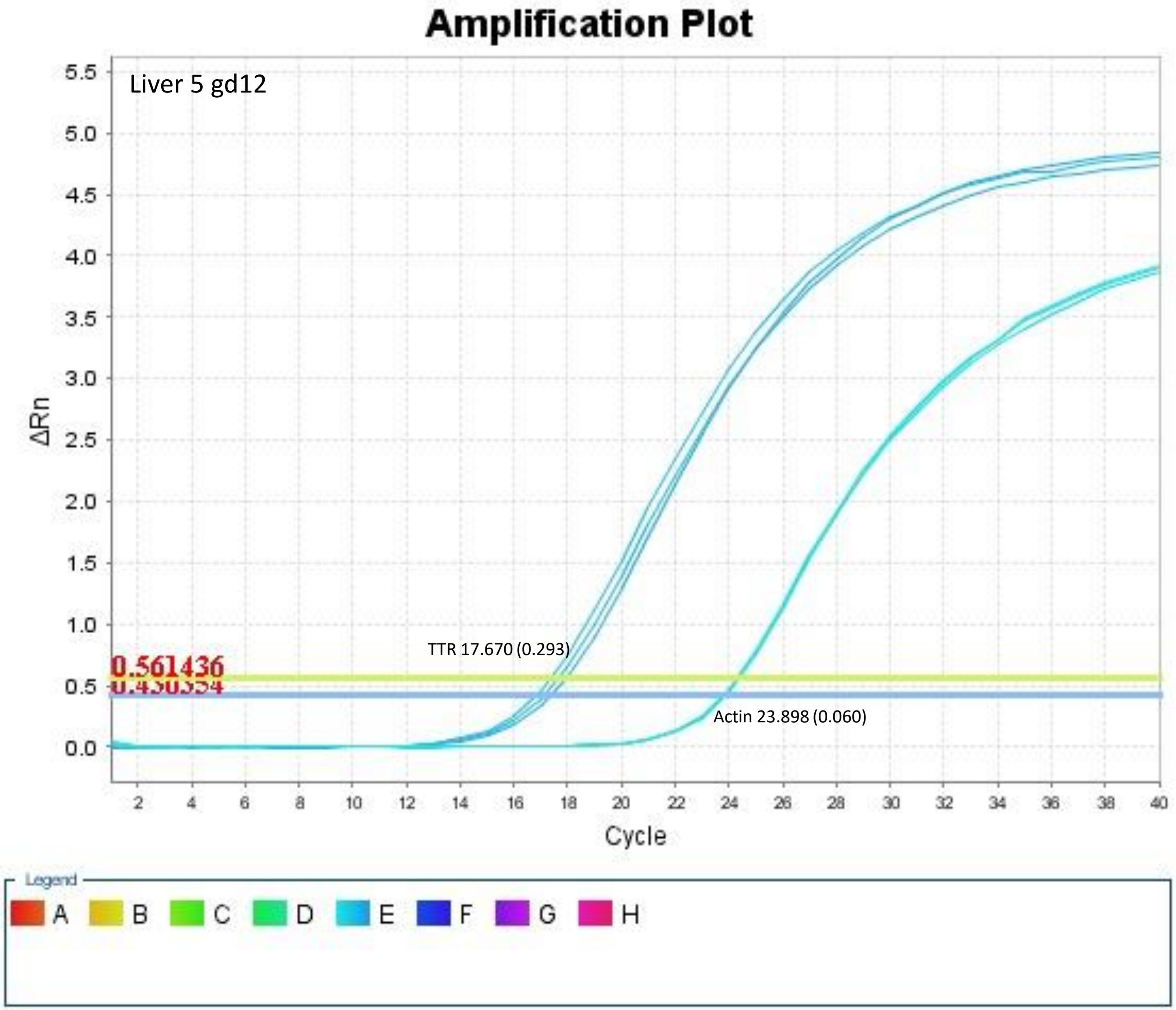

Amplification Plot
Liver 5 gd12
ΔRn
Cycle
0.561436
0.430354
TTR 17.670 (0.293)
Actin 23.898 (0.060)
Legend
A B C D E F G H

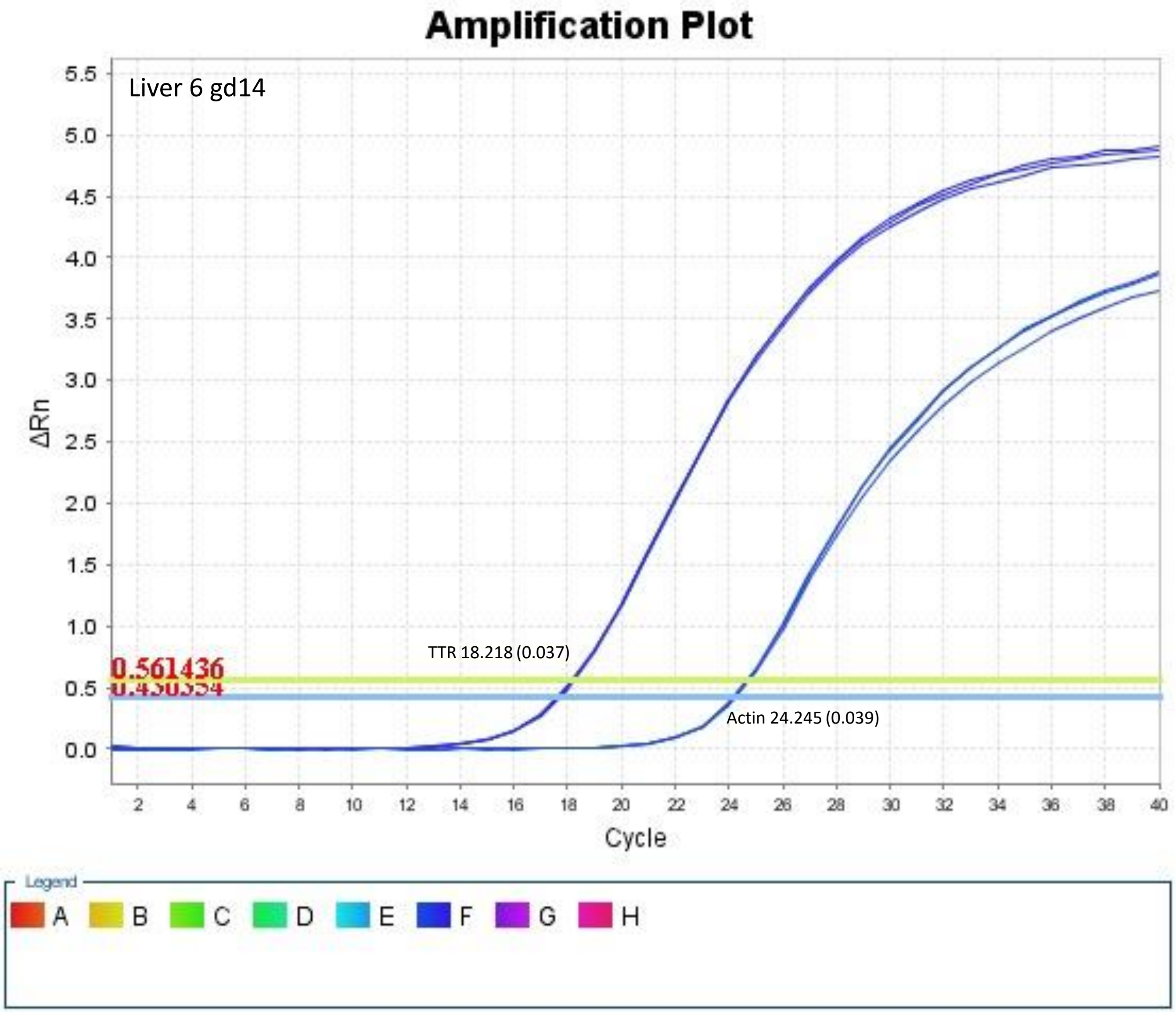

Amplification Plot
Liver 6 gd14
0.561436
TTR 18.218 (0.037)
Actin 24.245 (0.039)
ΔRn
Cycle
Legend
A B C D E F G H

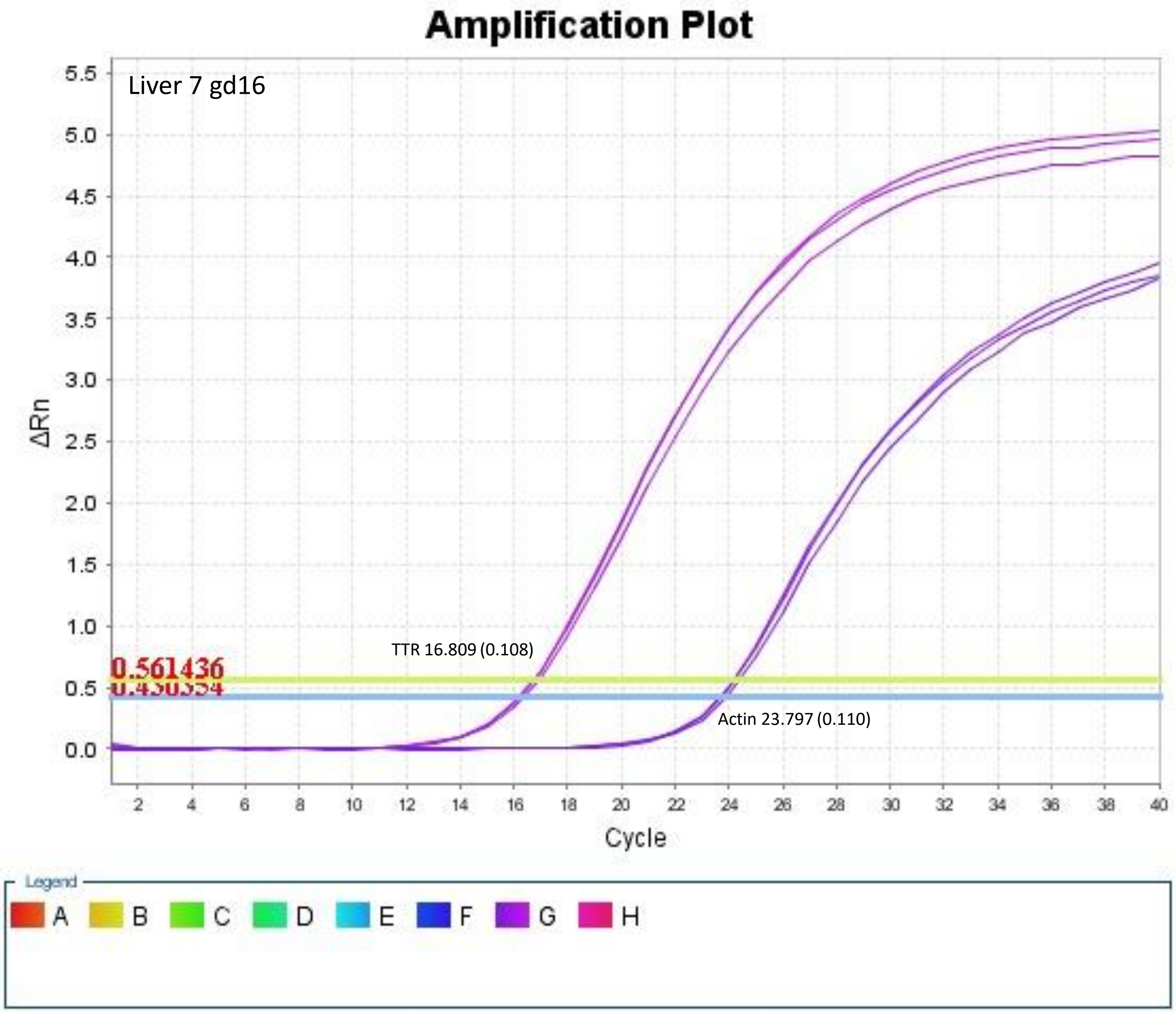

Amplification Plot
Liver 7 gd16
ΔRn
5.5
5.0
4.5
4.0
3.5
3.0
2.5
2.0
1.5
1.0
0.5
0.0
0.561436
TTR 16.809 (0.108)
Actin 23.797 (0.110)
2 4 6 8 10 12 14 16 18 20 22 24 26 28 30 32 34 36 38 40
Cycle
Legend
A B C D E F G H

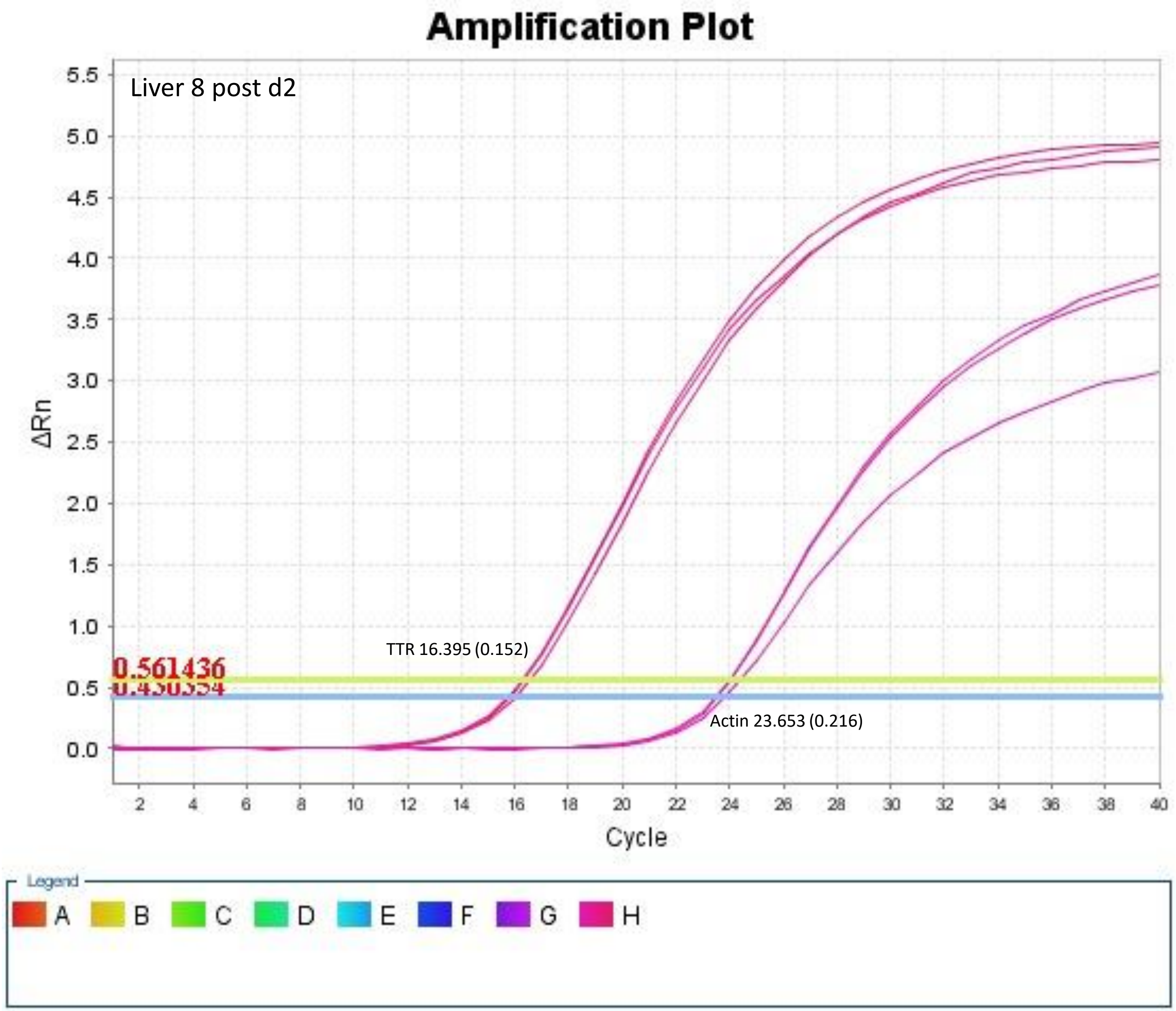

Amplification Plot
Liver 8 post d2
ΔRn
Cycle
0.561436
0.430354
TTR 16.395 (0.152)
Actin 23.653 (0.216)
Legend
A
B
C
D
E
F
G
H

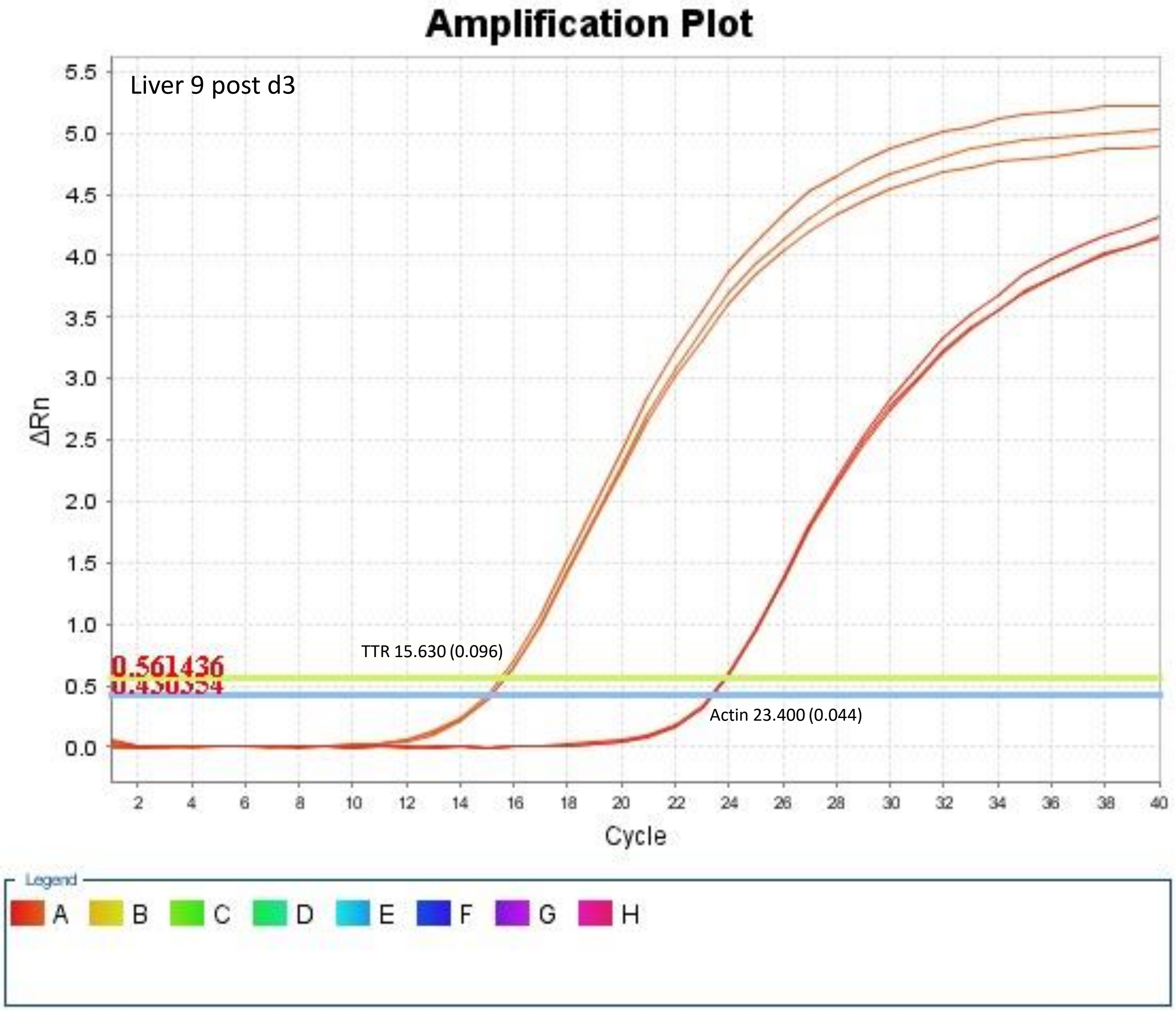

Amplification Plot
Liver 9 post d3
0.561436
TTR 15.630 (0.096)
Actin 23.400 (0.044)
ΔRn
Cycle
5.5
5.0
4.5
4.0
3.5
3.0
2.5
2.0
1.5
1.0
0.5
0.0
2
4
6
8
10
12
14
16
18
20
22
24
26
28
30
32
34
36
38
40
Legend
A
B
C
D
E
F
G
H

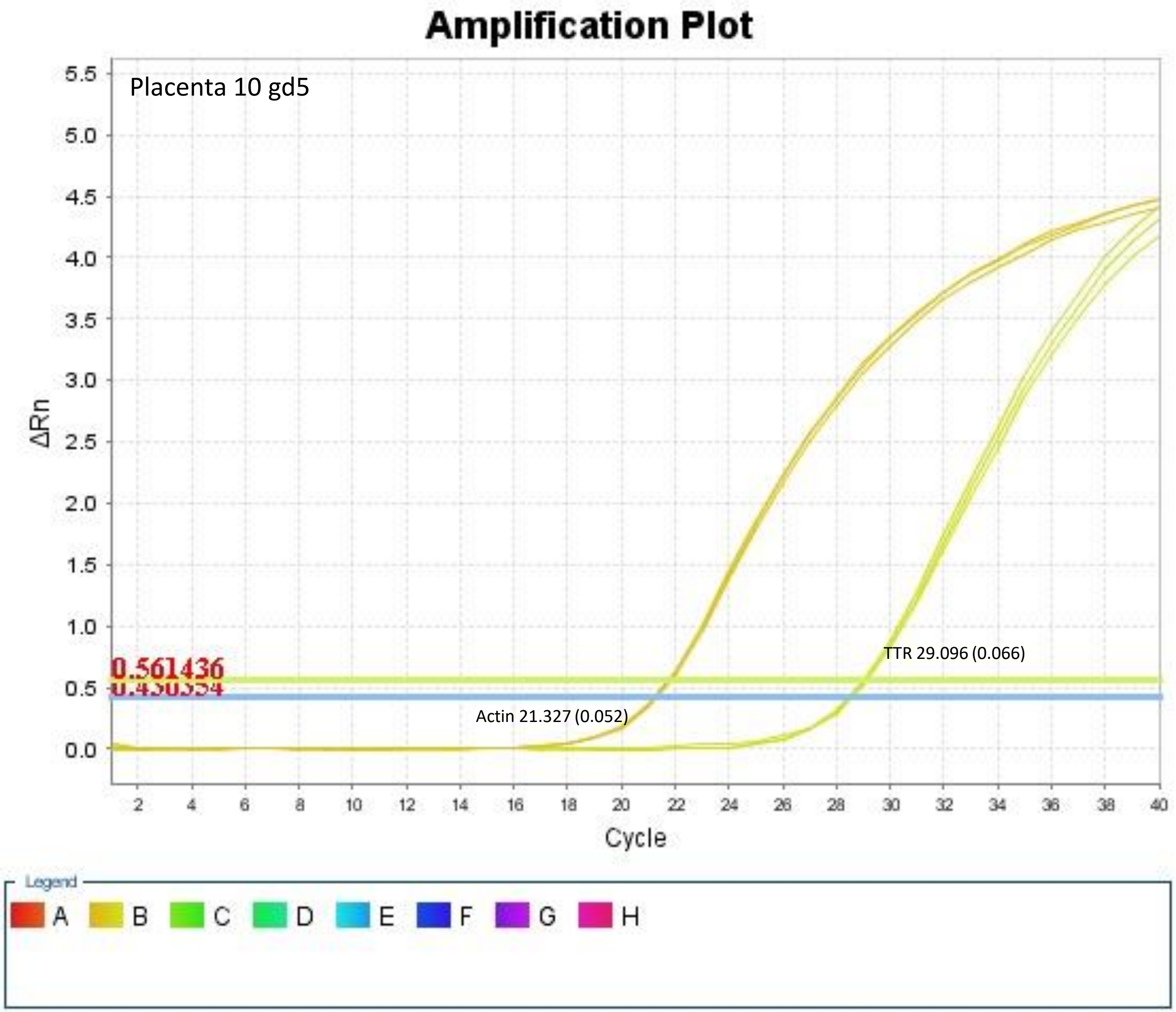

Amplification Plot
Placenta 10 gd5
ΔRn
5.5
5.0
4.5
4.0
3.5
3.0
2.5
2.0
1.5
1.0
0.5
0.0
0.561436
TTR 29.096 (0.066)
Actin 21.327 (0.052)
2
4
6
8
10
12
14
16
18
20
22
24
26
28
30
32
34
36
38
40
Cycle
Legend
A
B
C
D
E
F
G
H

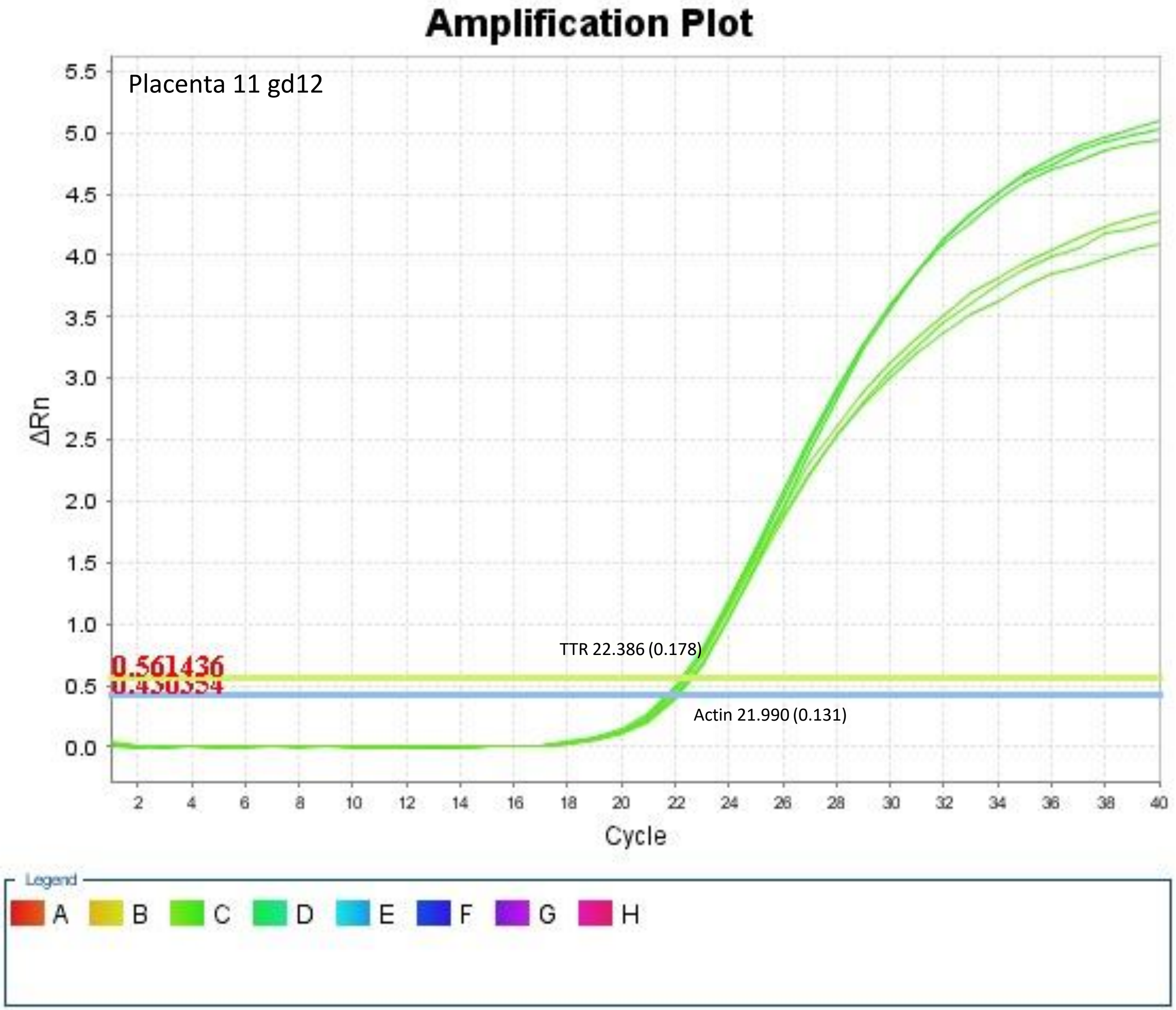

Amplification Plot
Placenta 11 gd12
ΔRn
5.5
5.0
4.5
4.0
3.5
3.0
2.5
2.0
1.5
1.0
0.5
0.0
0.561436
TTR 22.386 (0.178)
Actin 21.990 (0.131)
2
4
6
8
10
12
14
16
18
20
22
24
26
28
30
32
34
36
38
40
Cycle
Legend
A
B
C
D
E
F
G
H

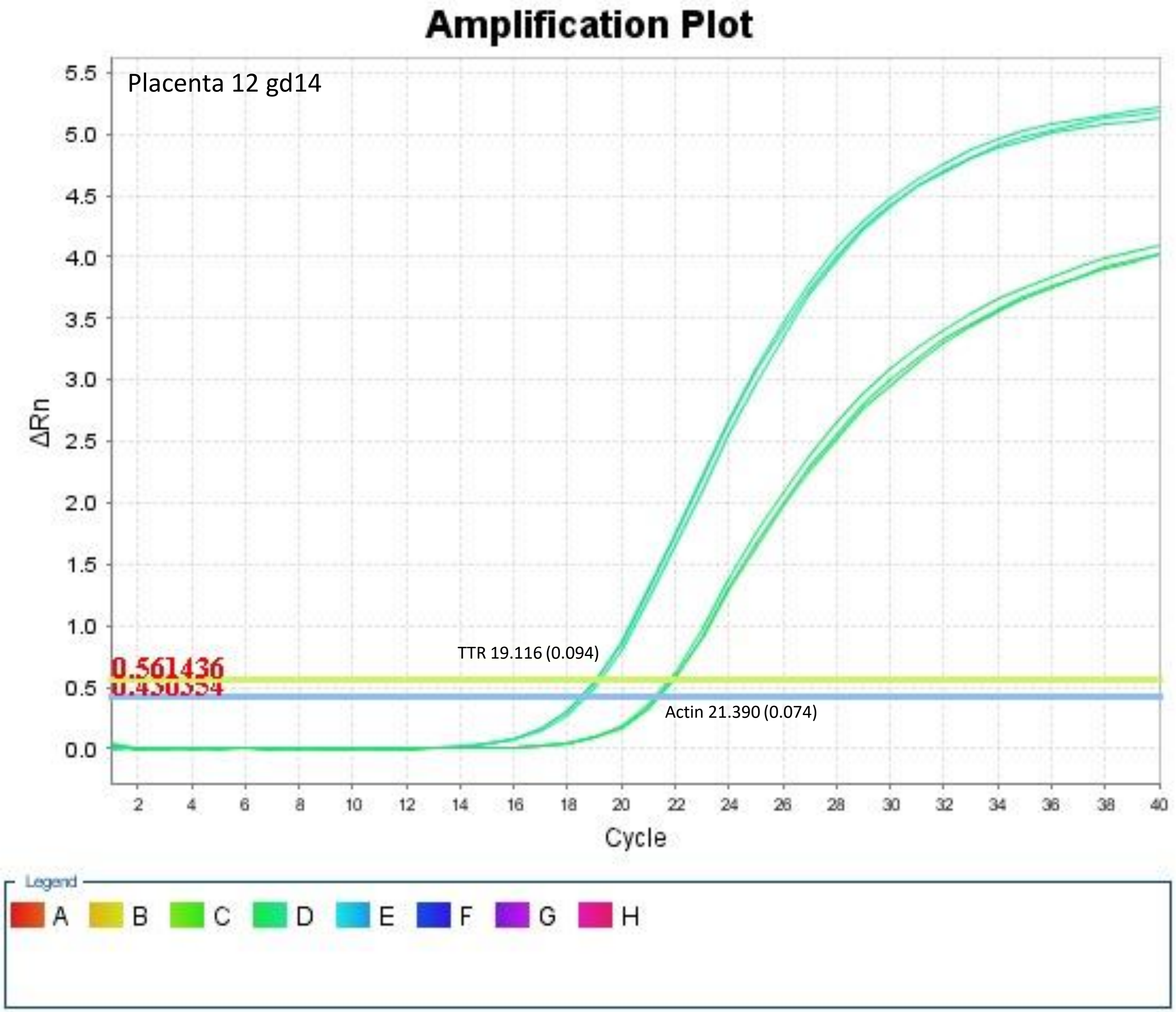

Amplification Plot
Placenta 12 gd14
ΔRn
Cycle
0.561436
TTR 19.116 (0.094)
Actin 21.390 (0.074)
Legend
A
B
C
D
E
F
G
H

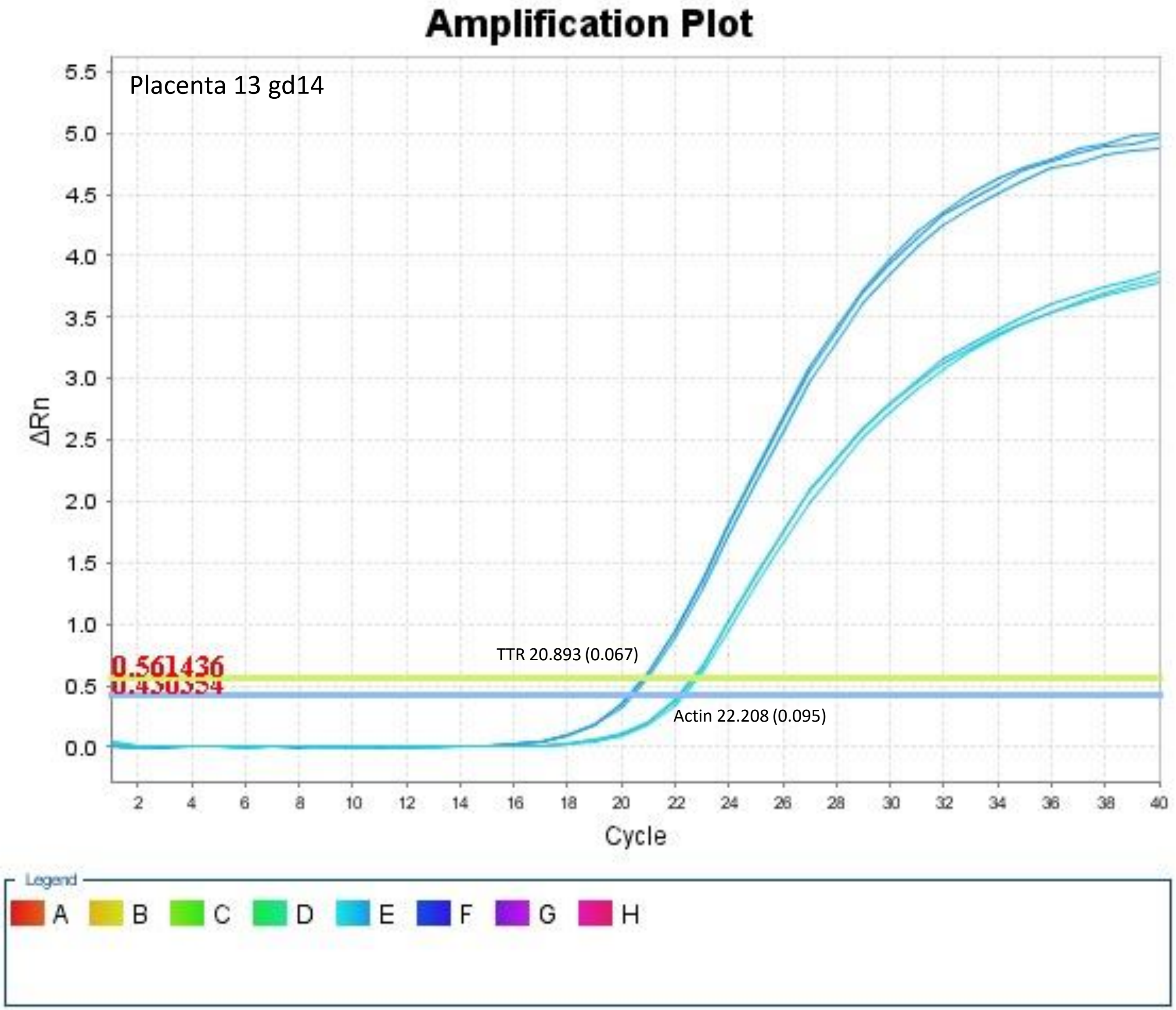
Amplification Plot
Placenta 13 gd14
ΔRn
Cycle
0.561436
TTR 20.893 (0.067)
Actin 22.208 (0.095)
Legend
A
B
C
D
E
F
G
H

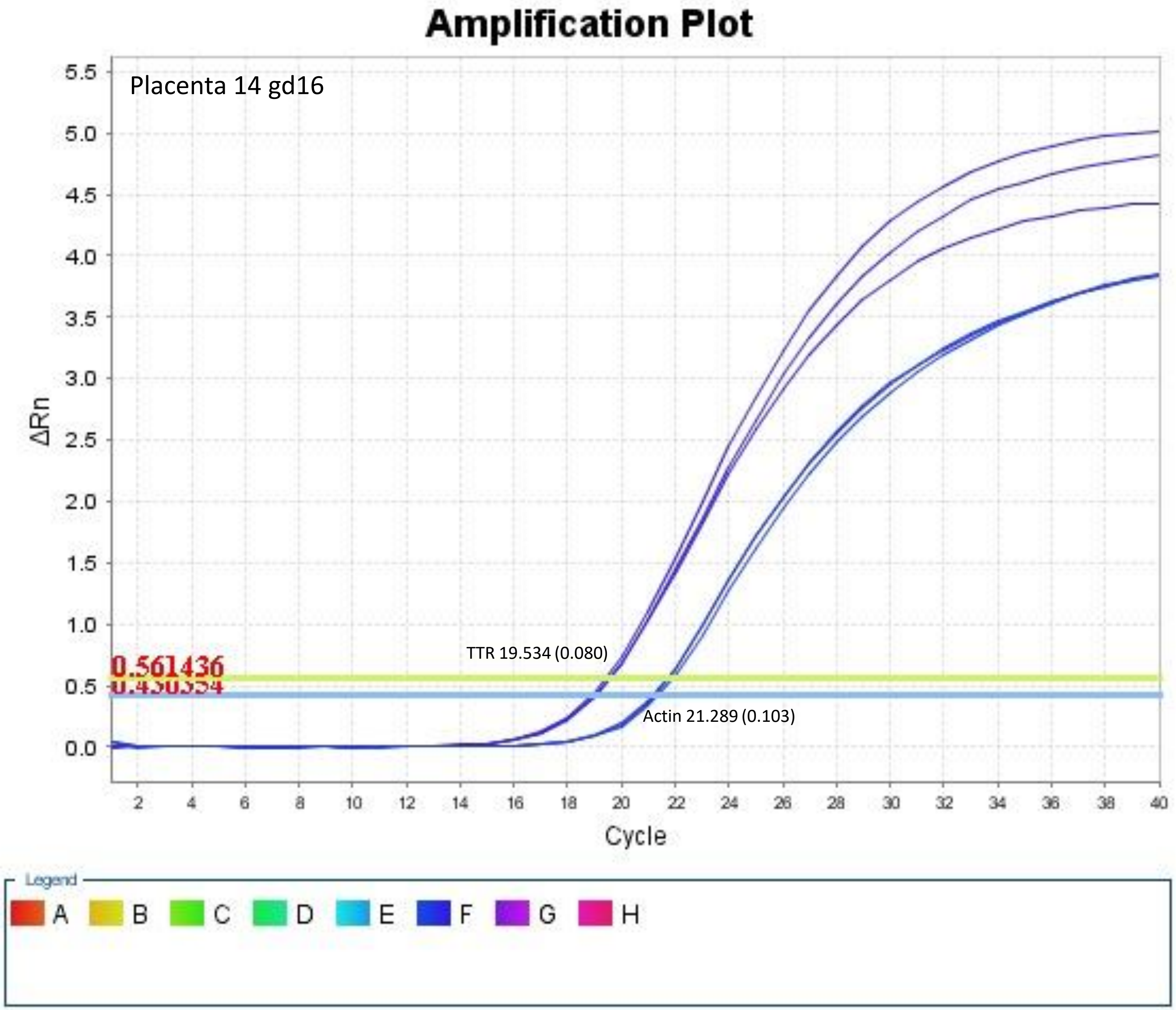
Amplification Plot
Placenta 14 gd16
ΔRn
Cycle
0.561436
TTR 19.534 (0.080)
Actin 21.289 (0.103)
5.5
5.0
4.5
4.0
3.5
3.0
2.5
2.0
1.5
1.0
0.5
0.0
2 4 6 8 10 12 14 16 18 20 22 24 26 28 30 32 34 36 38 40
Legend
A B C D E F G H

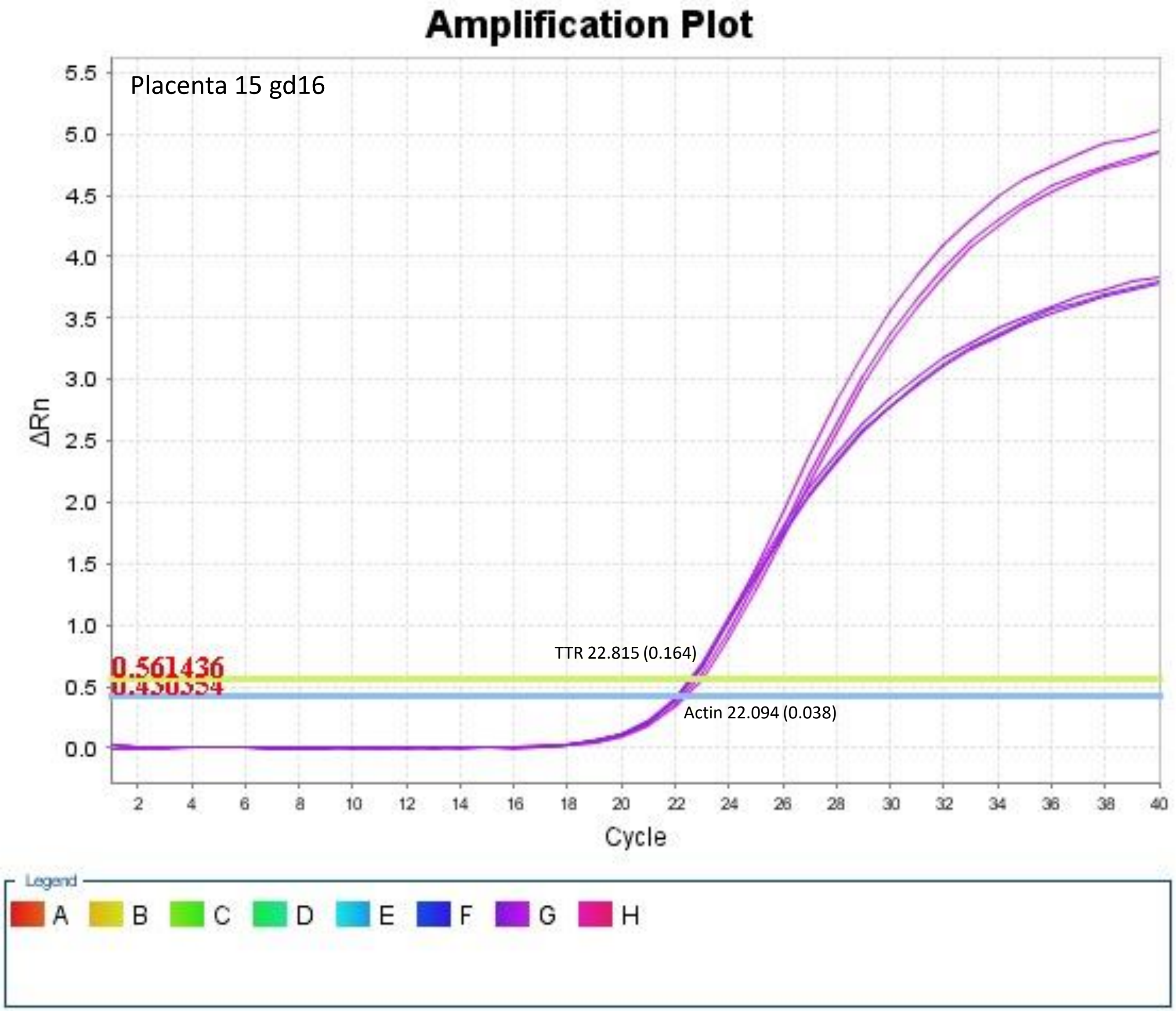
Amplification Plot
Placenta 15 gd16
0.561436
TTR 22.815 (0.164)
Actin 22.094 (0.038)
ΔRn
Cycle
Legend
A
B
C
D
E
F
G
H

Sample Name	Target Name	RQ	RQ Min	RQ Max	C_T	C_T Mean	C_T SD	Sample Name	Target Name	RQ	RQ Min	RQ Max	C_T	C_T Mean	C_T SD
Blank	TTR	1.000	0.733	1.365	31.186	31.225	0.034								
Blank	TTR				31.249										
Blank	TTR				31.240										
Blank	Actin				36.923	36.732	0.278								
Blank	Actin				36.860										
Blank	Actin				36.413										
Lv 2 non pg	TTR	2.159	2.029	2.298	17.747	17.787	0.048	Pl 10 gd5	TTR	0.000	0.000	0.000	29.021	29.096	0.066
Lv 2 non pg	TTR				17.774			Pl 10 gd5	TTR				29.121		
Lv 2 non pg	TTR				17.840			Pl 10 gd5	TTR				29.146		
Lv 2 non pg	Actin				24.435	24.404	0.029	Pl 10 gd5	Actin				21.313	21.327	0.052
Lv 2 non pg	Actin				24.379			Pl 10 gd5	Actin				21.386		
Lv 2 non pg	Actin				24.397			Pl 10 gd5	Actin				21.284		
Lv 3 non pg	TTR	2.869	2.735	3.009	17.490	17.498	0.038	Pl 11 gd12	TTR	0.017	0.013	0.021	22.232	22.386	0.178
Lv 3 non pg	TTR				17.465			Pl 11 gd12	TTR				22.581		
Lv 3 non pg	TTR				17.539			Pl 11 gd12	TTR				22.346		
Lv 3 non pg	Actin				24.547	24.525	0.021	Pl 11 gd12	Actin				21.887	21.990	0.131
Lv 3 non pg	Actin				24.522			Pl 11 gd12	Actin				22.137		
Lv 3 non pg	Actin				24.506			Pl 11 gd12	Actin				21.947		
Lv 4 gd5	TTR	1.841	1.608	2.109	17.057	17.161	0.098	Pl 12 gd14	TTR	0.106	0.093	0.122	19.074	19.116	0.094
Lv 4 gd5	TTR				17.174			Pl 12 gd14	TTR				19.050		
Lv 4 gd5	TTR				17.252			Pl 12 gd14	TTR				19.224		
Lv 4 gd5	Actin				23.627	23.548	0.073	Pl 12 gd14	Actin				21.433	21.390	0.074
Lv 4 gd5	Actin				23.483			Pl 12 gd14	Actin				21.433		
Lv 4 gd5	Actin				23.535			Pl 12 gd14	Actin				21.304		
Lv 5 gd12	TTR	1.649	1.183	2.298	17.385	17.670	0.293	Pl 13 gd14	TTR	0.055	0.048	0.062	20.971	20.893	0.067
Lv 5 gd12	TTR				17.654			Pl 13 gd14	TTR				20.861		
Lv 5 gd12	TTR				17.970			Pl 13 gd14	TTR				20.849		
Lv 5 gd12	Actin				23.961	23.898	0.060	Pl 13 gd14	Actin				22.317	22.208	0.095
Lv 5 gd12	Actin				23.842			Pl 13 gd14	Actin				22.142		
Lv 5 gd12	Actin				23.890			Pl 13 gd14	Actin				22.165		
Lv 6 gd14	TTR	1.435	1.352	1.523	18.186	18.218	0.037	Pl 14 gd16	TTR	0.074	0.064	0.086	19.600	19.534	0.080
Lv 6 gd14	TTR				18.208			Pl 14 gd16	TTR				19.446		
Lv 6 gd14	TTR				18.259			Pl 14 gd16	TTR				19.558		
Lv 6 gd14	Actin				24.230	24.245	0.039	Pl 14 gd16	Actin				21.408	21.289	0.103
Lv 6 gd14	Actin				24.217			Pl 14 gd16	Actin				21.238		
Lv 6 gd14	Actin				24.289			Pl 14 gd16	Actin				21.221		
Lv 7 gd16	TTR	2.792	2.353	3.313	16.933	16.809	0.108	Pl 15 gd16	TTR	0.013	0.011	0.016	22.843	22.815	0.164
Lv 7 gd16	TTR				16.736			Pl 15 gd16	TTR				22.639		
Lv 7 gd16	TTR				16.758			Pl 15 gd16	TTR				22.963		
Lv 7 gd16	Actin				23.922	23.797	0.110	Pl 15 gd16	Actin				22.133	22.094	0.038
Lv 7 gd16	Actin				23.755			Pl 15 gd16	Actin				22.056		
Lv 7 gd16	Actin				23.714			Pl 15 gd16	Actin				22.094		
Lv 8 post d2	TTR	3.390	2.529	4.543	16.271	16.395	0.152								
Lv 8 post d2	TTR				16.350										
Lv 8 post d2	TTR				16.565										
Lv 8 post d2	Actin				23.910	23.663	0.216								
Lv 8 post d2	Actin				23.564										
Lv 8 post d2	Actin				23.515										
Lv 9 post d3	TTR	4.803	4.269	5.403	15.520	15.630	0.096								
Lv 9 post d3	TTR				15.666										
Lv 9 post d3	TTR				15.702										
Lv 9 post d3	Actin				23.448	23.400	0.044								
Lv 9 post d3	Actin				23.360										
Lv 9 post d3	Actin				23.392										

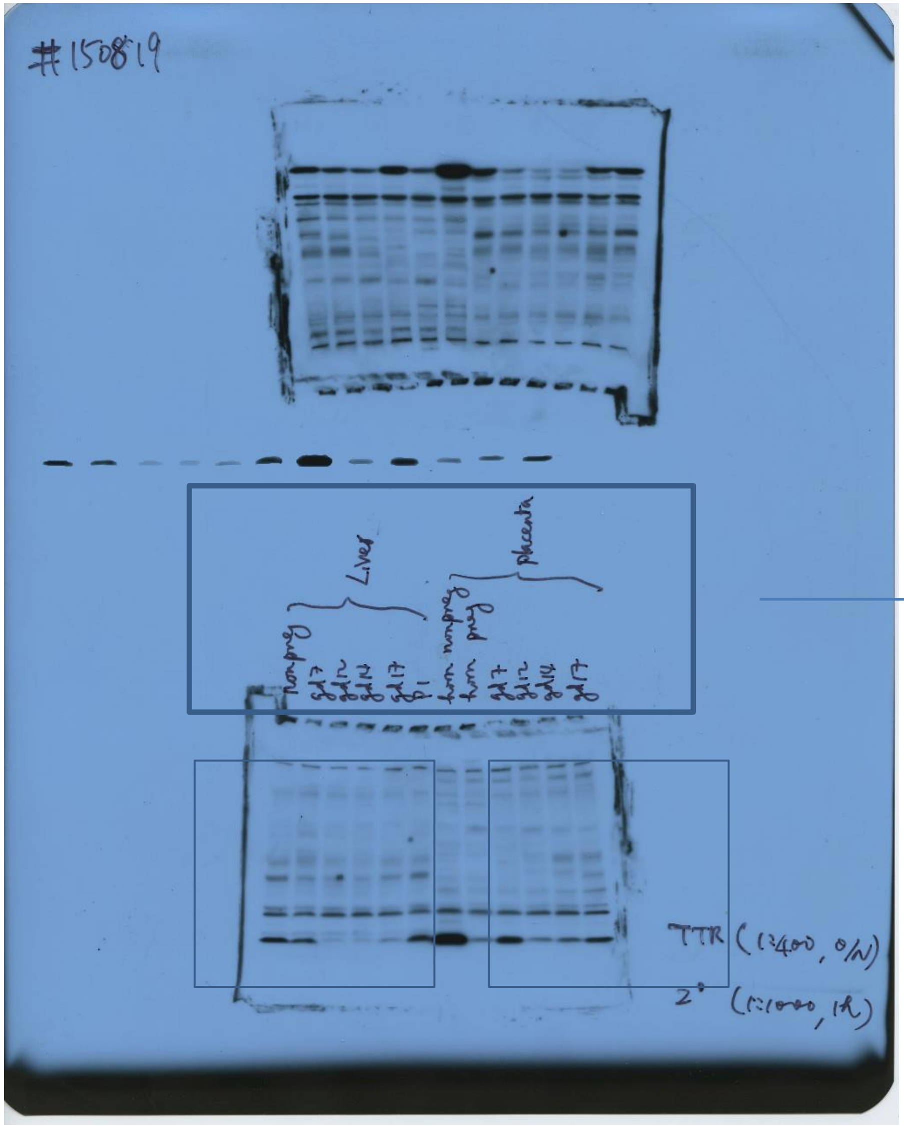

#150819
Liver
Placenta
TTR (1:400, O/N)
2° (1:1000, 1h)

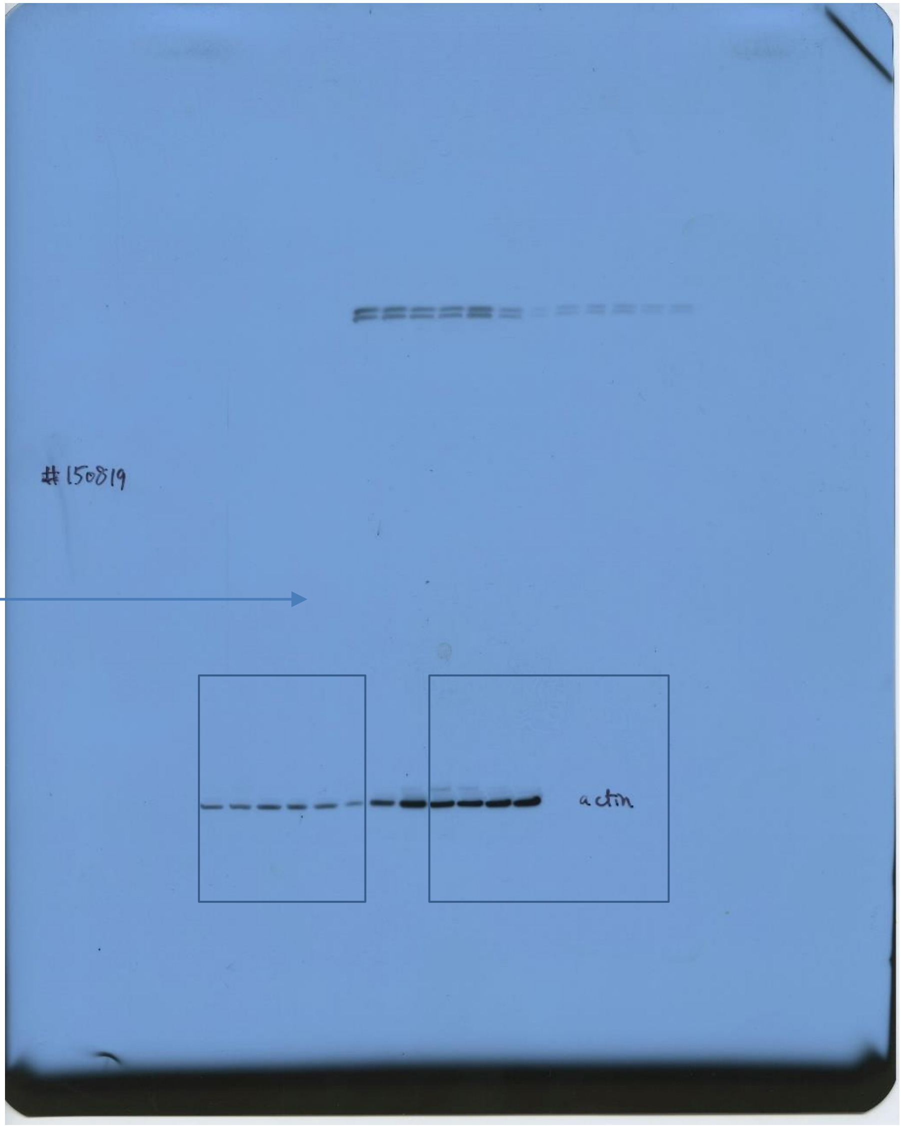

#150819
actin
